# Engineering an Endothelialized Vascular Graft: A Rational Approach to Study Design in a Non-Human Primate Model

**DOI:** 10.1371/journal.pone.0115163

**Published:** 2014-12-19

**Authors:** Deirdre E. J. Anderson, Jeremy J. Glynn, Howard K. Song, Monica T. Hinds

**Affiliations:** 1 Department of Biomedical Engineering, Oregon Health & Science University, Portland, OR, United States of America; 2 Division of Cardiothoracic Surgery, Oregon Health & Science University, Portland, OR, United States of America; Heart Research Institute, Australia

## Abstract

After many years of research, small diameter, synthetic vascular grafts still lack the necessary biologic integration to perform ideally in clinical settings. Endothelialization of vascular grafts has the potential to improve synthetic graft function, and endothelial outgrowth cells (EOCs) are a promising autologous cell source. Yet no work has established the link between endothelial cell functions and outcomes of implanted endothelialized grafts. This work utilized steady flow, oscillatory flow, and tumor necrosis factor stimulation to alter EOC phenotype and enable the formulation of a model to predict endothelialized graft performance. To accomplish this, EOC *in vitro* expression of coagulation and inflammatory markers was quantified. In parallel, in non-human primate (baboon) models, the platelet and fibrinogen accumulation on endothelialized grafts were quantified in an *ex vivo* shunt, or the tissue ingrowth on implanted grafts were characterized after 1mth. Oscillatory flow stimulation of EOCs increased *in vitro* coagulation markers and *ex vivo* platelet accumulation. Steady flow preconditioning did not affect platelet accumulation or intimal hyperplasia relative to static samples. To determine whether *in vitro* markers predict implant performance, a linear regression model of the *in vitro* data was fit to platelet accumulation data—correlating the markers with the thromboprotective performance of the EOCs. The model was tested against implant intimal hyperplasia data and found to correlate strongly with the parallel *in vitro* analyses. This research defines the effects of flow preconditioning on EOC regulation of coagulation in clinical vascular grafts through parallel *in vitro*, *ex vivo*, and *in vivo* analyses, and contributes to the translatability of *in vitro* tests to *in vivo* clinical graft performance.

## Introduction

Vascular grafts are an essential technology for treating cardiovascular disease, the dominant cause of death in our society. An estimated 416,000 bypass grafts procedures occurred in 2009 in the United States alone [1]. Bypass grafts represent an important intervention to treat vessel blockage, with autologous saphenous vein or mammary artery tissues remaining the standard treatment for the past 30 years. Artificial materials are used when native tissues are unavailable; while these materials have the mechanical properties to withstand suturing and arterial blood forces, their compliance mismatch encourages intimal hyperplasia and leads to graft failure. Many of these materials also lack the biologic capacity, predominantly due to the lack of endothelial-dependent regulatory functions, to integrate with the surrounding vessels leading to thrombosis. At small diameters (<5 mm), tissue thickening leads to a decrease in the graft lumen resulting in occlusive failure of the graft. As reviewed elsewhere, various biologic materials are being used or developed for vascular grafts, but current clinical products still have considerable limitations in mechanical integrity, patient wait time, immune rejection, and ultimately patency [2].

Given the limited ability of an artificial graft to fully endothelialize in humans [3], a tissue-engineered graft or endothelialized biomaterial may be a more desirable implant when a native graft is unavailable. Endothelial cells tightly control vascular homeostasis. As reviewed previously, endothelial cells produce a multitude of transmembrane glycoproteins and released factors which regulate coagulation, thrombin generation, and platelet activity [4]–[6]. Of particular interest to this work are activated factor X (FXa), which converts prothrombin to thrombin, and activated protein C (APC), which leads to a downregulation of thrombin. The activation of APC is enhanced by endothelial transmembrane glycoproteins, thrombomodulin (TM) and endothelial protein C receptor (EPCR). Factor Xa results from the complex of transmembrane tissue factor (TF) with factor VIIa, while endothelial cell release of tissue factor pathway inhibitor (TFPI) inhibits both FXa and the TF-factor VIIa complex. Endothelial cells regulate platelet activation through the production of the enzyme endothelial nitric oxide synthase (eNOS) resulting in nitric oxide release and the expression of transmembrane CD39 [5]. In addition to thrombosis and hemostasis, endothelial cells are central regulators of inflammation [7], [8]. Intercellular adhesion molecule-1 (ICAM), vascular cell adhesion molecule-1 (VCAM), and platelet endothelial cell adhesion molecule (PECAM) are endothelial transmembrane proteins that facilitate leukocyte adhesion and migration in inflammation. Combined, these endothelial functions cooperatively regulate the processes of hemostasis, inflammation, and intimal hyperplasia. Endothelialization of vascular grafts has been demonstrated to improve vascular graft patency by properly regulating these processes and facilitating integration with the surrounding tissue [9]–[12]. Biochemical or mechanical, particularly fluid shear stress, stimulation can be used to alter and improve endothelial cell phenotype. Steady, laminar fluid flow with physiological shear stress leads to endothelial cell elongation and alignment in the direction of flow, decreased cell proliferation, decreased monocyte adhesion, and downregulation of endothelial regulated immunogenicity. Alternatively, low or disturbed flow leads to a lack of cell alignment and an increase in immunogenicity [6]. The phenotype of the elongated endothelial cell improves thromboprotective function, suggesting that preconditioning an endothelialized graft may result in superior *in vivo* performance.

To obtain autologous endothelial cells for graft endothelialization would require an invasive harvest, while implantation of endothelial cells from allogenic or xenogenic sources would require continuous immune suppression. Given the limited supply of attainable mature vascular endothelial cells, endothelial outgrowth cells (EOCs), which are obtained from a simple blood draw and expand rapidly in culture, are an attractive autologous cell source for an engineered endothelium. This descendent of endothelial progenitor cells has seen widespread use in the past decade. Recent work has begun to characterize EOCs under various shear conditions and found similar results to the well-established shear responses seen with mature endothelial cells [13]–[16]. EOCs exhibited the most thromboprotective and immunoprotective phenotype after stimulation with steady fluid shear stress and the most thromboprone phenotype after stimulation with oscillatory shear stress [14]. EOCs were also responsive to tumor necrosis factor (TNF), a potent proinflammatory cytokine [13], [15], [17]. Stimulation with these mechanical or biochemical stimuli should elicit a large range of EOC phenotypes.

A critical limitation for all research on vascular graft development is the lack of testing modalities that translate into improved clinical function. *In vivo* testing in relevant animal models will be an essential final step in determining the performance of any graft, but it is neither cost-effective nor feasible for initial studies. Therefore, many researchers test a panel of coagulation or inflammation markers on their cell source and/or biomaterial of choice. As reviewed previously [18], many studies have attempted to mimic *in vivo* graft performance by examining the response of a single blood component (e.g., platelets or leukocytes), but these simplified models are unlikely to accurately represent the *in vivo* performance of the graft. Even the majority of implant models lack the strength to generate clinical translatability. Small animal models, which often employ a short (1–2cm length) end-to-end graft, spontaneously endothelialize and do not model human vascular graft responses [19].

To address these limitations we used two, well-established baboon models: an *ex vivo* shunt model and an *in vivo* end-to-side graft. The shunt model uses whole blood at physiologic arterial flow conditions to recapitulate coagulation factor transport dynamics. Furthermore, this model characterizes vascular graft thrombogenicity without anticoagulants to more accurately reflect how grafts will perform in patients who will not be given anticoagulants long-term [20]–[25]. The baboon aorto-iliac implant model of an end-to-side 4mm diameter vascular graft is designed to most closely relate to the clinical use of vascular grafts in humans, thereby maximizing the translatability of this research [19], [26]. This model does not spontaneously endothelialize, but rather shows an ingrowth of smooth muscle cells and endothelial cells at a rate of 0.2 mm/day [26]. For both these models, we examined EOCs on a clinically-relevant graft material, expanded polytetrafluoroethylene (ePTFE). While this material did not address the compliance mismatch plaguing artificial vascular grafts, the grafts represent the current gold-standard artificial material. By performing *in vitro* analyses of thrombosis and inflammation in parallel with *ex vivo* or *in vivo* responses in these non-human primate models, we identified the *in vitro* metrics that best correlate to platelet accumulation and performance in the body. Using both biochemical and mechanical methods to alter EOC phenotype we aimed to generate correlations with broad applicability. Specifically, we tested the EOCs in vascular grafts under static culture conditions, steady and oscillatory fluid shear stress preconditioning, and TNF stimulation. These stimuli generated a large range of phenotypic responses which facilitated the development of a linear regression model correlating *in vitro* EOC responses to platelet or tissue ingrowth in vascular grafts. We hypothesized that 1) steady fluid shear stress would lead to a more anticoagulant and anti-inflammatory EOC phenotype *in vitro*, reduced platelet accumulation *ex vivo*, and reduced intimal hyperplasia *in vivo*, and 2) *in vitro* metrics of the EOC functions would correlate with *ex vivo* platelet accumulation. Identification of these metrics now provides an essential starting point for all vascular graft research to more rapidly improve clinical vascular grafts. This multifaceted research determined not only the performance of this clinically-relevant, cell-based therapy *in vivo*, but also the ability to predict *in vivo* and *ex vivo* responses based on *in vitro* analyses.

## Materials and Methods

### Experimental Design

These studies were designed to examine EOC coagulation and inflammatory markers *in vitro*, platelet and fibrinogen accumulation *ex vivo*, and endothelialized graft performance *in vivo* on EOC-seeded ePTFE grafts. Oscillatory or steady fluid flow preconditioning, as well as TNF treatment, was performed on EOC-seeded grafts to elicit a range of EOC phenotypes. *In vitro* analyses were performed on EOC-seeded grafts prepared and treated in parallel with samples used for the *ex vivo* shunt studies and the *in vivo* implant studies to determine correlations between *in vitro* analyses and *ex vivo* or *in vivo* performance. Samples were tested *in vitro* for cell coverage using fluorescent microscopy and DNA quantity, for FXa and APC production using biochemical assays, and for gene expression using qRT-PCR. In parallel, a baboon, femoral, arteriovenous shunt model, which utilizes whole blood lacking anticoagulation with radiolabeled platelets and fibrinogen, was used to quantify platelet and fibrin accumulation on the cell-seeded grafts. Alternatively, intimal hyperplasia was quantified on end-to-side aorto-iliac bypasses using a blind analysis on cell-seeded grafts implanted contralaterally to untreated controls in a baboon implant. These parallel analyses of preconditioning EOC-seeded vascular grafts were performed to determine correlations resulting from *in vitro* metrics of EOC phenotype. Raw data can be found in the supporting files (S1 File).

### EOC isolation, culture, and flow pretreatment

Late-outgrowth, baboon EOCs were derived from the mononuclear cells of peripheral blood as described previously [27]. Cell phenotype was confirmed after passage 1 by selecting for PECAM positive cells using Dynabeads (Invitrogen) according to the manufacturer's protocol. EOCs were maintained in endothelial growth media-2 (Lonza) with 10% fetal bovine serum on collagen-coated tissue culture plastic and used at passage 5 in these studies. Graft lengths of 50 or 70 mm of ePTFE with a 4 mm internal diameter (Gore-Tex, W.L. Gore and Associates, Inc.) were connected to silicone tubing, maintaining a smooth lumen. Grafts were then coated with 4 mg/mL bovine collagen I (MP Biomedicals), and seeded with EOCs in a custom biochamber, as described previously [28]. After 24hrs, the media was switched to a higher viscosity flow media to mimic blood viscosity: 15% solution of 100 g/L dextrose and 320 g/L dextran from Leuconostoc mesentreroides (Sigma, molecular weight = 48,000–90,000) in endothelial basal media (Lonza) with 5% fetal bovine serum, 1% Penicillin-Streptomycin, 1% L-glutamine, and 0.1% epidermal growth factor. Samples were either stimulated with 10 dyn/cm^2^ steady shear flow, 0±10 dyn/cm^2^ oscillatory shear flow at 1 Hz, or kept as static cultures for an additional 24hrs.

### *In vitro* assessments

Grafts for *in vitro* analyses were prepared in parallel with grafts used for *in vivo* or *ex vivo* studies. All analyses were performed on all *in vitro* samples.

#### Fluorescent staining

EOC-seeded graft segments (about 10 mm in length) were stained for F-actin with AlexaFluor 568 phalloidin and nuclear co-stained with DAPI, as described previously [28]. For PECAM staining, graft sections were incubated with mouse anti-human PECAM (Dako) overnight at 4°C, washed in PBS with 0.1% BSA, and stained with AlexaFluor 568-tagged goat anti-mouse IgG (Invitrogen). Stained sections were mounted with ProLong Gold antifade reagent (Invitrogen) and imaged using an Olympus FV1000 confocal microscope and Fluoview software.

#### Total DNA

The DNA content of EOC-seeded graft segments was measured with the PicoGreen dsDNA assay according to the manufacturer's protocol (Invitrogen) and normalized by graft surface area after measurements with digital calipers.

#### qRT-PCR

Cells were lysed from a 25–35 mm long section of ePTFE, and RNA was isolated from the lysate using an RNeasy mini isolation kit (Qiagen). Samples were reverse transcribed with SuperScript III Reverse Transcriptase (Invitrogen) according to the manufacturers' protocols. Gene expression was quantified using Platinum SYBR Green and ROX reference dye (Invitrogen) with in-house designed primers at 250 nM concentration (S1 Table). All i*n vitro* samples were tested for a panel of coagulation and inflammation genes, specifically, CD39, EPCR, TM, eNOS, ICAM, VCAM, PECAM, TF, and TFPI. Glyceraldehyde 3-phosphate dehydrogenase (GAPDH) served as a housekeeping gene and ddct values were determined by comparing treated samples to static controls. Data were transformed by subtracting 1 from any positive 2^-ddct^ values and taking the negative inverse of any negative 2^-ddct^ values and adding 1. This resulted in a continuous, linear data set and is referred to as fold change, where the static values are 0. Positive values show an increase from paired static samples, while negative values indicate a decrease from the static control.

#### Functional assays

EOC-seeded graft segments approximately 3 mm in length were incubated with 5 nM thrombin (Haematologic Technologies) and 100 nM Protein C (Haematologic Technologies) for 60 min at 37°C to generate APC. Refludan (Berlex) or hirudin was used to inactivate the thrombin, and a chromogenic substrate (1 mM S-2366, Chromogenix) was used to quantify APC generation by comparing it to an APC standard (Haematologic Technologies). Graft segments for FXa quantification approximately 3 mm in length were incubated with 20 nM factor VIIa (Enzyme Research Laboratories) and 200 nM factor X (Enzyme Research Laboratories) for 60 min at 37°C. Cold EDTA (5 nM) was used to stop the reaction, and 0.67 mM Spectrozyme FXa (American Diagnostica) was used to measure FXa generation compared to a FXa standard. Absorbance at 405 nm was measured every 20 s for 20 min and the maximum slope over 10 points used to quantify APC or FXa. Data were normalized by the graft segment area.

### Animal care and ethics

Male baboons (Papio anubis) were cared for and housed at the Oregon National Primate Research Center (ONPRC) at Oregon Health & Science University. All the experiments described here were reviewed and approved (approval numbers IS00002496 and IS00002092) by the Oregon Health & Science University West Campus Institutional Animal Care and Use Committee according to the “Guide for the Care and Use of Laboratory Animals” prepared by the Committee on Care & Use of Laboratory Animals of the Institute of Laboratory Animal Resources, National Research Council (International Standard Book, Number 0-309-05377-3, 1996). The ONPRC is a Category I facility. The Laboratory Animal Care and Use Program at the ONPRC is fully accredited by the American Association for Accreditation of Laboratory Animal Care (AAALAC), and has an approved Assurance (#A3304-01) for the care and use of animals on file with the Office for Protection from Research Risks at the National Institutes of Health (NIH). The Division of Animal Resources (DAR) is composed of highly trained veterinarians, clinicians, and medical technologists. All nonhuman primates are housed in indoor or indoor/outdoor facilities. Nonhuman primates are routinely fed commercially prepared primate feed milled within the past 90days. This feed is supplemented daily with fruit and special diets prepared in the ONPRC diet kitchen. Fresh, potable water is provided by the municipal water district via automatic water systems. The nonhuman primates are provided with daily enrichment including (1) toys (in cage at all times and rotated every 2 weeks) and (2) devices (foraging manipulanda) on the outside of the cage, also changed every 2 weeks. The devices are filled with trail mix, grain, and/or produce at least 3 days per week. The nonhuman primates receive produce on days the devices are not filled, (3) TV/radio on a weekly rotation schedule, (4) pair housing/grooming contact (as much as possible), (5) positive reinforcement training, and (6) cognitive enrichment (computer tablets, such as Kindles). Steps are taken to ameliorate suffering according to the Weatherall Report “The use of non-human primates in research.” During experimentation, the animals are monitored for behavior, appetite, and stool quantity. The technician reports any abnormalities on a daily observation sheet, which is checked daily by a member of the clinical services unit and the behavioral services unit. Any notations by the technician about abnormalities with the animal are addressed by both the clinical services unit and the behavioral services unit.

For all surgical procedures, animals were anesthetized with 1–3% isofluorane delivered at 1–2 L/min in 100% oxygen after induction by ketamine (10–20 mg/kg intramuscularly) and Telazol (3–5 mg/kg intramuscularly). During the procedure the DAR staff monitored pulse rate, body temperature, blood pressure, ECG, oxygen saturation, and exhaled CO_2_. Adequacy of anesthesia was additionally assessed by observable spontaneous movements, and physical, circulatory, and respiratory responses to surgical stimuli. Animals received a short acting opioid pain medication 3 times/day for 3days post-surgery and a long acting pain medication once/day for 48–72hrs. If it was determined by the veterinary staff that pain medication was needed after the prescribed regime, permission is given by the research staff for it to be prescribed to the animal as needed. After surgery the nonhuman primates were monitored for visible signs of infection or fever, mobility or swelling of the lower limbs, lameness, cold toes, plate limbs, as well as bleeding from surgical sites (anemia, reduced hematocrit); however, this was not observed during these studies, and no animals were terminated prior to study endpoint. At the implant study endpoint, to remove the implanted vascular grafts and analyze the results, the animals were euthanized according to the AVMA Guidelines on Euthanasia June 2007. For euthanasia, the animals were given ketamine 20 mg/kg followed by phenobarbitol (25 mg/kg) and exsanguination.

### *Ex vivo* shunt studies

Shunt studies were performed on a femoral arteriovenous shunt as described previously [28]. Briefly, autologous platelets and homologous fibrinogen were radiolabeled with indium-111 and iodine-125, respectively, and infused into the baboon. An *ex vivo* femoral arteriovenous shunt loop was established in the absence of anti-coagulant and anti-platelet therapies. EOC-seeded ePTFE grafts within custom chambers were connected to the shunt loop with blood flow controlled at 100 ml/min using a clamp downstream of the graft. This flow rate corresponds to an average of 10 dyn/cm^2^ wall shear stress. Platelet deposition was measured for 1 hr using a gamma camera with 5 min exposure increments (static n = 26, steady n = 11, oscillatory n = 9). Fibrinogen accumulation was quantified at the study endpoint after complete decay (>10 half-lives) of the indium-111 (static n = 24, steady n = 9, oscillatory n = 9).

### Implant studies

Aorto-iliac bypass ePTFE grafts were implanted bilaterally in juvenile male baboons, weighing 12–16 kg under anesthesia described above. After a midline incision, heparin (100 units/kg) was given and the aorta occluded with atraumatic microvascular clamps. Grafts were positioned between the distal aorta and common iliac artery, at the iliac bifurcation. Static (n = 7) or steady flow-pretreated (n = 4) EOC-seeded samples were implanted contralaterally to an untreated ePTFE control sample. Grafts were seeded using autologous EOCs. Anastomoses were constructed (end-to-side) using continuous 7–0 polypropylene sutures. This procedure is similar to the standard surgical placement of such grafts in humans and produces progressive proliferative lesion formation at anastomotic sites. After 4wks, grafts were pressure fixed in 10% formalin and explanted. After overnight fixation in 10% formalin, the grafts were sectioned, dehydrated, embedded in paraffin, and stained with hematoxylin and eosin using standard histological procedures. The mid-point of the each anastomoses was analyzed for tissue ingrowth on the ePTFE grafts. The area of intimal hyperplasia was measured from the edge of the anastamosed artery to a 2 mm length into the graft using Image J. Trained viewers quantified these regions while blinded to which samples were controls or treated. The area of ingrowth was averaged across each 2 mm ingrowth section of the anastomosis. Intimal hyperplasia was only analyzed for patent anastomoses. While other groups have found differences between tissue ingrowth in the proximal and distal anatomoses [29], [30], the historic data with our animal model for both bare and cell-seeded end-to-side vascular grafts have shown no difference between the two anastomoses [31]–[34]. Therefore, data from this work were averaged across proximal and distal locations and analyzed to evaluate the hypothesis of endothelial cell flow preconditioning.

### Correlations and linear regression model development

Single linear regression models were developed to correlate all *in vitro* data from each individual data point (FXa generation, APC generation, DNA quantity, CD39 gene expression, EPCR gene expression, TM gene expression, TF gene expression, TFPI gene expression, eNOS gene expression, ICAM gene expression, VCAM gene expression, and PECAM gene expression) to the parallel *ex vivo* data point (platelet accumulation at 60 min). The R^2^ coefficient/value and p-value were used to examine the strength and significance of these single variable regressions. To create a predictive model of platelet accumulation, additional samples were added to challenge the data set and create a more robust model. In addition to the oscillatory flow, steady flow, and static samples described previously, a group of samples of EOC-coated grafts were stimulated with 100 U/mL TNF (R&D Systems) for 4hrs prior to testing *in vitro* and *ex vivo*. This potent cytokine elicited a proinflammatory EOC phenotype, increasing the range of cell responses and potentially broadening the applicability of the developed regression model. When using gene data in the correlation analysis, values were converted to a linear scale using 2^-dCt^. When a single factor correlation's p-value was less than 0.25, that factor was considered potentially important to develop the regression model. Factors that were strongly correlated to one another were not included in the same regression model. Models which resulted in strong correlations (R^2^>0.6) were tested using the *in vitro* control data collected parallel to implanted grafts to determine the applicability of the model to predict *in vivo* healing responses.

### Statistical analysis

All data are presented as mean ± standard deviation. Sample numbers varied throughout this experiment, partially because static control samples were always run parallel to flow- or TNF-treated samples; however, n≥3 for all treatment groups. Outlier analysis, defined as greater than 3 standard deviations away from the mean, was performed, but points were only removed when data collection indicated an error may have occurred. For example, samples were excluded if actin staining on parallel *in vitro* control samples indicated insufficient cell coverage on the graft. Unless otherwise stated, data were analyzed with a 1-way analysis of variance (ANOVA) to determine changes between treatment groups. Significance was indicated when p<0.05, and a Tukey's *post hoc* test was performed. A 2-way ANOVA was performed on the platelet accumulation data with factors of treatment and time. Data were checked for assumptions of ANOVA. Specifically, a Levene's test was performed to check for unequal variances, since sample sizes were not equal. Occasionally the data showed a slight skewedness (non-normality) in the distribution, and in these cases, a Box-Cox transformation was performed [35]. The same statistical analysis (ANOVA with a Tukey's *post hoc*) was run on the transformed data. Since there were no changes in the statistically-significant results, these transformations are not presented.

In the linear regression analysis, significance was determined using a main effects test for the overall regression and a 2-tailed t-test for the individual coefficients of the model. The standardized coefficients (beta) were determined in SPSS (IBM corp.) and indicate the scaled variables to compare the dominance of each of the variables compared to the others.

## Results

### Hemodynamic treatments altered the phenotype of EOCs

**Figure 1:**
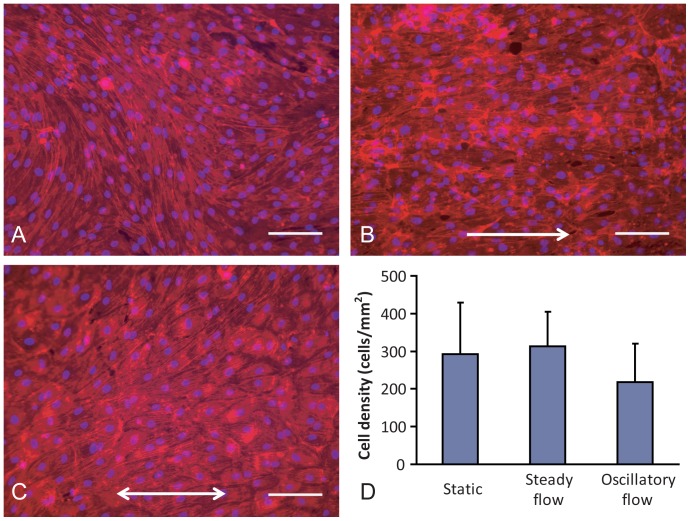
Cell coverage and quantification. Representative fluorescent images of EOCs seeded on ePTFE grafts for 24hrs followed by 24hrs of continued static culture (**A**), 10 dynes/cm^2^ steady fluid shear stress (**B**), or 0±10 dynes/cm^2^ at 1 Hz oscillatory shear stress (**C**). EOCs without flow conditioning had either a cobblestone morphology or random cell alignment. Steady shear stress imparted an elongated cell morphology, with cells aligning in the direction of flow. Oscillatory shear stress induced a rounded morphology. Scale bar equals 100 µm. Arrows indicate the direction of flow stimulation. Actin filaments were stained red with nuclei in blue. Cell density (**D**), as determined by the PicoGreen assay for DNA quantification, indicated no significant differences between treatment groups. ANOVA, p = 0.32. N≥6.

**Figure 2:**
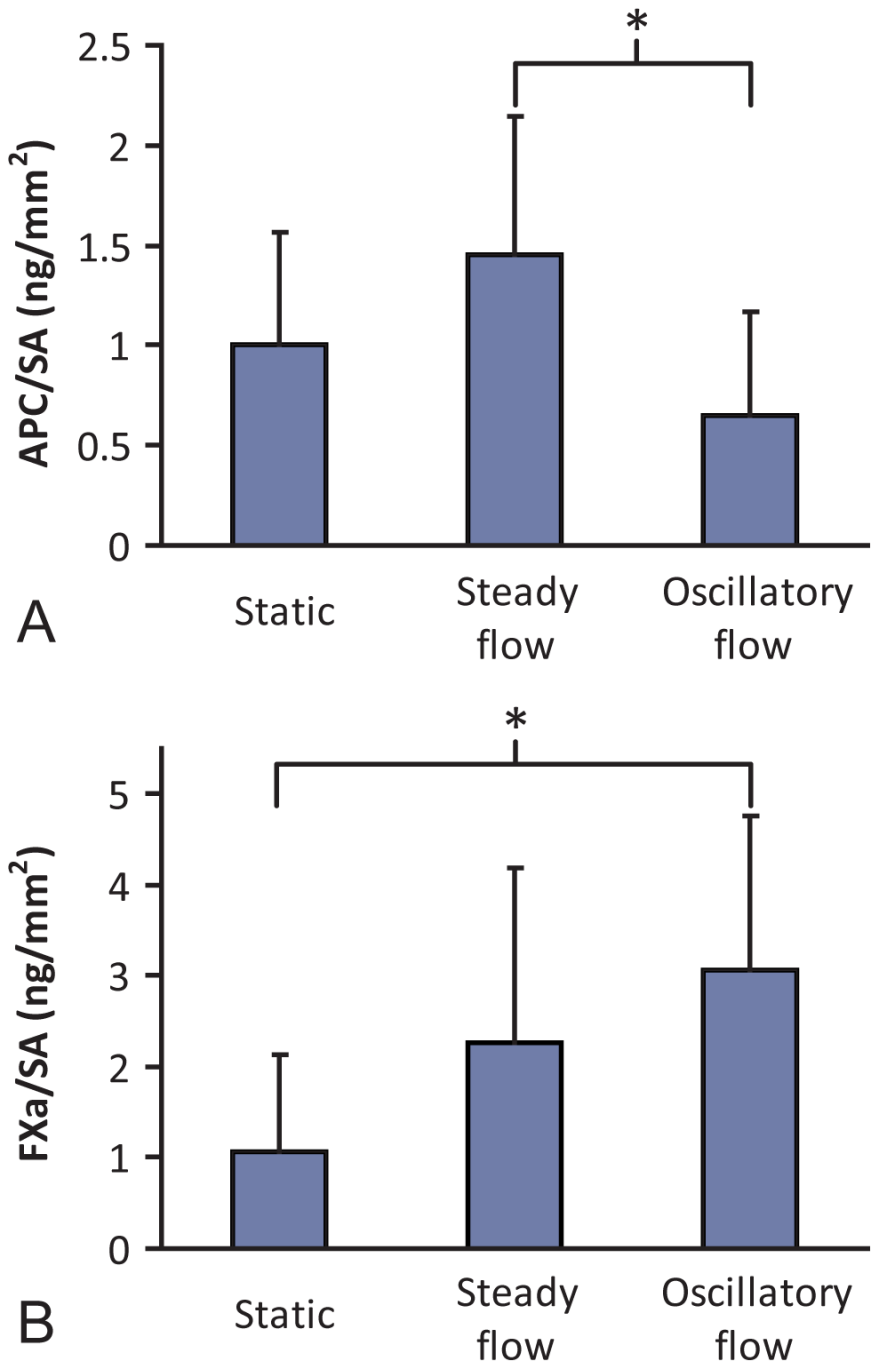
*In vitro* functional data. Quantification of APC and FXa production by EOCs seeded onto ePTFE grafts and stimulated with steady fluid shear stress, oscillatory shear stress, or continued static culture for 24hrs after 24hrs of initial seeding. APC production per surface area (SA) was significantly higher in the steady flow treated sample when compared to the oscillatory flow treated sample (**A**) (*p = 0.05). FXa production per surface area was significantly higher in the oscillatory treated sample compared to the no flow control (**B**) (*p = 0.01). ANOVA, N≥6.

EOC phenotype in response to no flow (static), mean arterial (steady) flow, and disturbed (oscillatory) flow was characterized. In the absence of flow, the morphology of EOCs on ePTFE vascular grafts was typical of mature endothelial cells with elongation occurring in somewhat random orientation (Fig. 1A and S1A Fig.), but with the majority of the alignment seen in the circumferential direction (S2 Fig.). Steady shear-treated EOCs (Fig. 1B and S1B Fig.) consistently showed elongation of the cells and alignment of the EOCs' actin cytoskeleton to the direction of fluid shear stress. Oscillatory shear-stimulated EOCs (Fig. 1C and S1C Fig.) exhibited a rounded morphology. Despite these large morphological changes, cells were retained under all testing conditions and the DNA quantity (Fig. 1D) was unchanged between all treatment types. The hemodynamic conditions altered the expression of genes related to thrombin generation, platelet attachment, and inflammation relative to no-flow controls (Table 1). Steady shear stress treatment caused a significant increase in eNOS and CD39 gene expression by the EOCs compared to the oscillatory shear stress treatment. Additionally, the hemodynamic conditions altered production of the two key components by which endothelial cells regulate thrombin generation, APC and FXa. Steady flow pretreatment of the EOCs significantly increased APC production (Fig. 2A) compared to oscillatory shear stress pretreatment. Oscillatory shear-treated EOCs produced significantly more FXa (Fig. 2B) compared to the EOCs which remained under static conditions. Combined, these data show the prothrombotic phenotype elicited in the EOCs from oscillatory flow preconditioning.

**Table 1:** *In vitro* EOC gene expression grouped by primary role in vascular homeostasis.

	FXa Pathway		APC Pathway		Inflammation			Platelet	
	TF	TFPI	TM	EPCR	ICAM	VCAM	PECAM	eNOS*	CD39*
Steady shear	1.49±3.50	-0.53±0.70	1.56±3.23	1.18±0.73	-1.25±1.27	0.25±2.94	0.61±1.33	0.73±0.80	1.13±0.72
Oscillatory shear	1.34±1.44	0.07±0.57	-0.34±1.15	0.51±0.68	0.40±1.80	-0.44±1.67	-2.19±2.44	-1.13±1.77	-1.30±1.33

Fold increase/decrease of EOC gene expression for flow pretreated samples compared to static. Values greater than 0 indicate an increase from paired static samples while values less than 0 indicate a decrease from static pair. ANOVA, *p<0.05. N≥7.

### Hemodynamic treatment of EOCs altered platelet and fibrin accumulation

**Figure 3:**
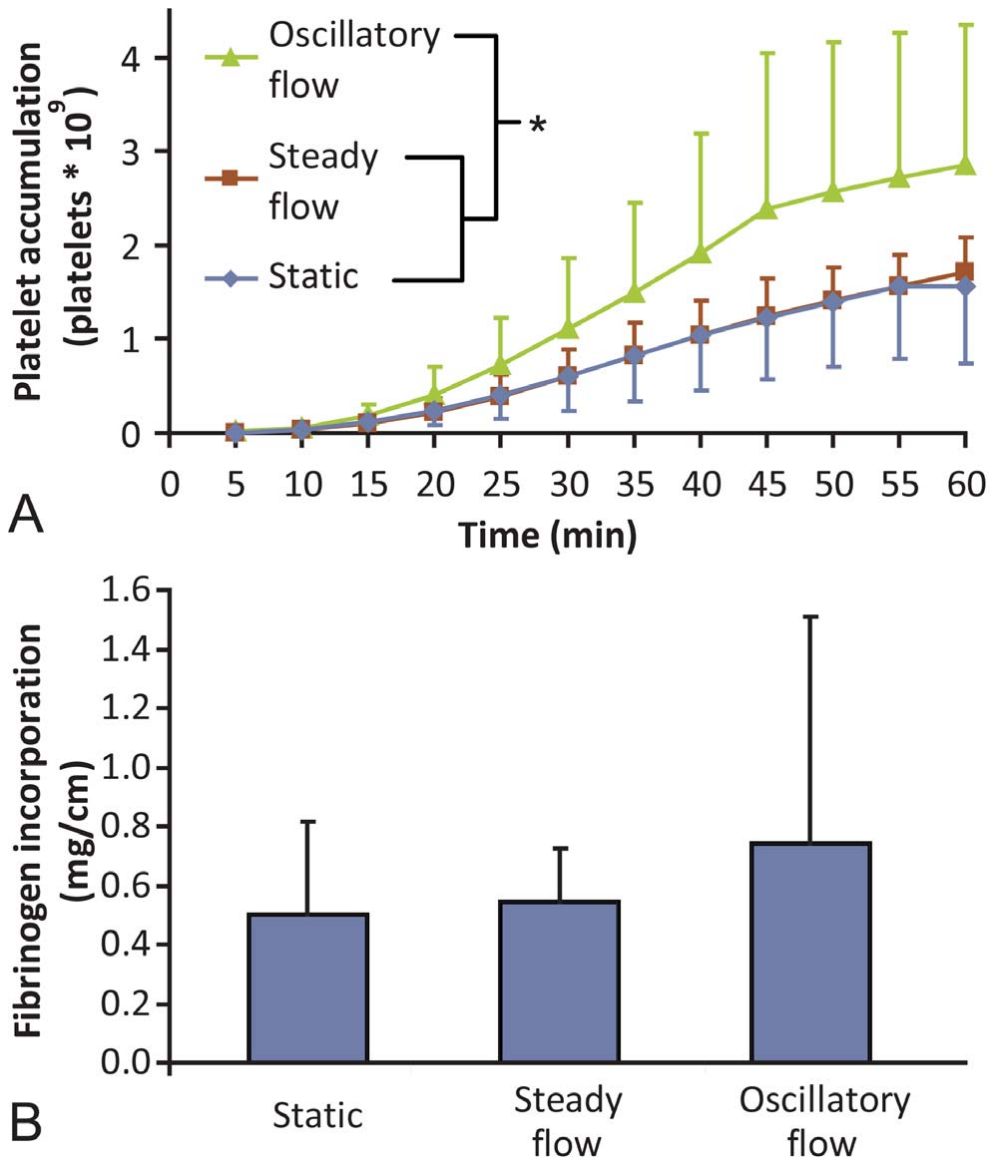
*Ex vivo* platelet and fibrin data. EOC-seeded grafts in the *ex vivo* shunt loop had significantly more platelet deposition over 60 min when pretreated with oscillatory shear stress compared to either steady shear stress or no flow preconditioning (**A**) (*p = 0.01). Fibrinogen incorporation (**B**) also trended higher in oscillatory treated samples, but was not statistically different (p = 0.13). In samples that occluded before 60 min, platelet count at the time of occlusion was included through 60 min for the purposes of illustration. This occurred in three of the oscillatory samples. ANOVA, N≥9.

To determine the effect of EOC phenotype on the antithrombotic properties of the endothelialized vascular grafts, flow preconditioned endothelialized grafts were placed in a well-established non-human primate model of vascular thrombosis. Platelet accumulation in the absence of anticoagulants was significantly higher in the oscillatory flow-stimulated sample compared to both the steady flow and static samples (Fig. 3A). Steady flow conditioning of EOC-coated samples did not alter the amount of platelet accumulation compared to the static conditioned grafts. Fibrinogen incorporation was not significantly different between the three treatment groups (Fig. 3B); however, the average amount of fibrinogen in the oscillatory treatment samples was higher compared to static and steady flow samples.

### Steady flow treatment did not alter vascular healing response on ePTFE grafts

**Figure 4:**
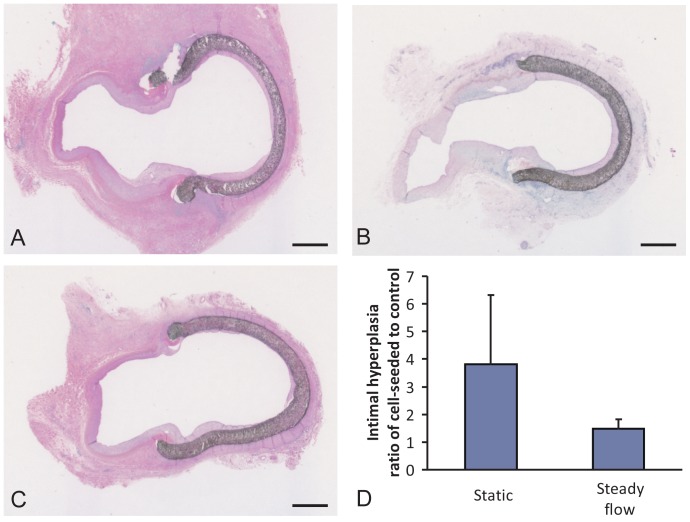
Baboon implant results. Representative hematoxylin and eosin histology stains are shown for each of the treatment groups: static, EOC-seeded grafts (**A**), EOC-seeded grafts pre-treated with steady 10 dynes/cm^2^ fluid shear stress for 24hrs prior to testing (**B**), and bare, unmodified ePTFE (**C**). Scale bar equals 1 mm. Intimal hyperplasia quantification after 4wks in a baboon graft model is shown for the cell-seeded samples as a ratio to their internal, unmodified control (**D**). ANOVA, p = 0.18. N≥4.

To test whether steady flow treatment of EOC endothelialized vascular grafts would improve long term vascular responses, autologous EOC-coated vascular grafts with contralateral control ePTFE grafts (without cells) were implanted in a non-human primate, end-to-side aorto-illiac bypass model. All animals survived this study; however, in one implant, all four anastomoses (control and static cell-seeded) were closed at explant. Two additional anastomoses from different animals both from cell-seeded distal sections without flow pretreatment were also not patent. Representative histology images from the distal anastomoses of each treatment group are shown in Fig. 4. To account for the animal to animal variation, the amount of intimal hyperplasia on the endothelialized vascular grafts was normalized to the contralateral ePTFE control graft. The flow pre-treatment of the EOCs did trend toward lower intimal hyperplasia, although the difference was not statistically different (p = 0.162, Fig. 4D).

### Linear regression model results

**Figure 5:**
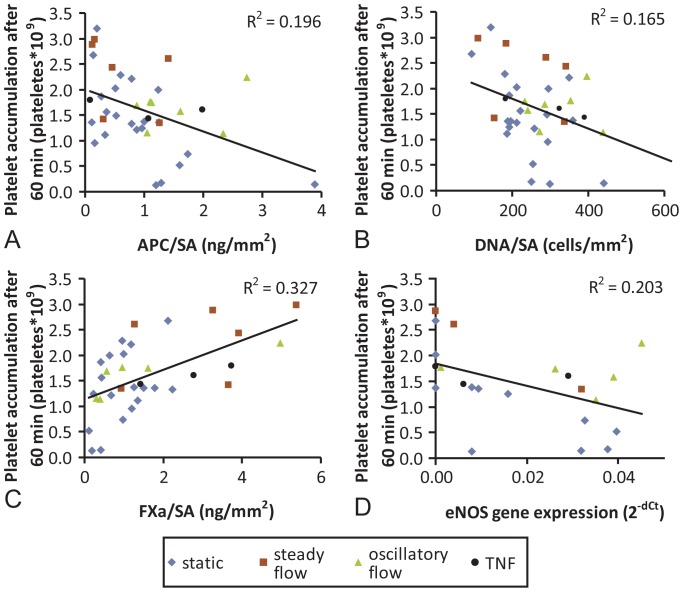
Single factor model regressions. Platelet deposition after 1 hr in the *ex vivo* shunt loop correlated to the parallel *in vitro* assessments of APC/SA (**A**), DNA/SA (**B**), FXa/SA (**C**), and gene expression of eNOS (**D**). While none of the single factors resulted in strong correlations (R^2^>0.6), these four were all significant correlations (p<0.05).

To determine the potential for *in vitro* assessments of EOC function on vascular grafts to predict platelet accumulation, we developed a linear regression model. The linear regression analysis was performed on single *in vitro* variables for all individual data points against the corresponding total platelet accumulation at 60 min using the mechanically- and biochemically-modified EOC phenotypes (Fig. 5 and S2 Table). APC generation, FXa generation, DNA per surface area, and eNOS gene expression showed significant correlations (p<0.05); however, their R^2^ values were still quite low, suggesting the need for multiple factors in generating a regression model. Variables that resulted in highly not significant (p>0.5) correlations were ICAM, VCAM, and TF gene expression.

**Figure 6:**
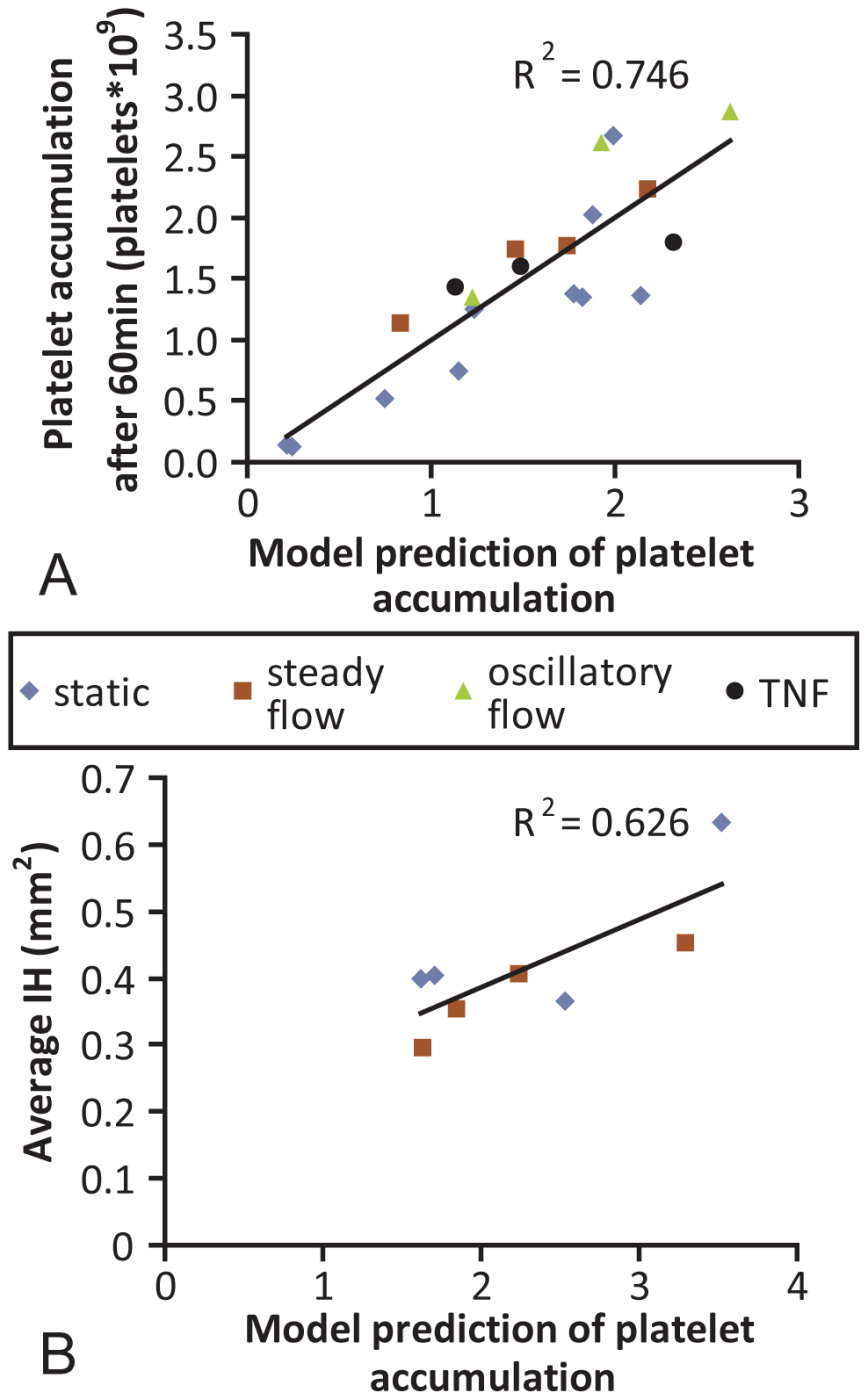
Multifactor linear regression model. Following analysis of 19 multifactorial models (Table S4), the strongest correlation model included EOC generation of FXa and expression of genes for eNOS and CD39. The predicted platelet attachment by the model compared to the actual platelet accumulation had an R^2^ of 0.746 (**A**). The amount of predicted platelet attachment had a strong correlation to the degree of anastamotic intimal hyperplasia (**B**).

Nineteen models, having between 2 and 4 variables, were tested based on the significance of the individual correlations when the TNF data were included. The R^2^ values of these combined models ranged from 0.187 to 0.746 (S3 Table). The highest R^2^ value resulted from combining the factors FXa, eNOS, and CD39 (Fig. 6A and S4 Table). The model equation was:
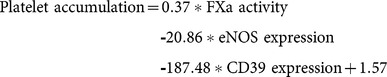



To determine the utility of the linear regression model predicting *in vivo* outcomes of implanted grafts, the model was applied to the data set from the *in vitro* grafts constructed in parallel to the implant grafts. From the regression model, the platelet accumulation was predicted and compared to the *in vivo* outcome of anastomotic intimal hyperplasia. The FXa/eNOS/CD39 model correlated to intimal hyperplasia measurements at 4wks after graft implant with an R^2^ value of 0.626 and a significance of 0.019 (Fig. 6B).

## Discussion

Endothelialization is expected to be a critical component for improving the patency of small diameter, artificial, vascular grafts. The past decade has seen a substantial increase in the interest in EOCs, and endothelial progenitor cells in general, for multiple therapeutic applications; however, considerable work is still needed to determine the applicability of these cells for vascular devices. The objectives of these studies were (1) to determine the ability of flow preconditioning to alter hemostatic regulation of EOCs in a clinically-relevant graft large animal model and (2) to correlate *in vitro* thrombogenic and inflammatory markers of EOCs with *ex vivo* and *in vivo* outcomes. Completing these objectives improves our understanding of EOCs, a clinically-feasible source for autologous endothelial cells, and suggests predictive metrics for vascular graft success in future implant designs.

There is a considerable body of scientific work that has examined the response of mature endothelial cells to fluid shear stress, but only a handful of recent publications have explored the response of EOCs to these same stresses. Based on previous work with endothelial cells and EOCs [13]–[15], [36]–[39], it was expected that the application of steady flow would lead to a more anticoagulant and anti-inflammatory EOC phenotype *in vitro*, reduced platelet accumulation *ex vivo*, and reduced intimal hyperplasia *in vivo*. When compared to oscillatory shear stress, this hypothesis was largely true. EOC gene expression of the anti-platelet molecules CD39 and eNOS were significantly increased after steady shear compared to oscillatory shear. EOCs produced significantly more APC *in vitro* when preconditioned with 10 dyn/cm^2^ steady fluid shear stress compared to oscillatory, while oscillatory shear increased FXa production compared to static controls. Oscillatory flow preconditioning also increased platelet accumulation when compared to static or steady flow samples. These results corresponded well to previous work, which used the same conditioning on EOCs seeded on tissue culture plastic. Ankeny *et al.* [14] measured increases in EOC gene expression of thromboprotective genes (including eNOS and CD39) but saw no change in inflammatory, TF, or TFPI genes for oscillatory stimulation compared to steady shear. Oscillatory flow consistently produced a procoagulant phenotype in the EOCs, confirming EOC sensitivity to disturbed hemodynamic conditions.

An unexpected observation was the lack of differences between EOCs under steady fluid shear stress compared to static conditions. There were no significant differences between steady flow and static preconditioning for APC or FXa generation. The platelet accumulation on these samples was nearly identical over 60 min, and no differences were seen in fibrinogen incorporation. Ahmann *et al.* [13] observed no change in ICAM or VCAM gene expression between static and steady shear stress, but most previous work observed changes in thrombosis markers with steady flow. Previous work on EOCs demonstrated an increase in TM protein [36] and gene expression [36], [37], [40], an increase in TFPI gene expression [36], and an increase in APC production [36], [40] with steady shear stress compared to static. NO production [13], eNOS gene expression [13], [15], [36], [37], [39], and eNOS protein production [15] by EOCs have all been observed to increase under steady shear stress compared to no flow conditions. Platelet binding also decreased with steady flow preconditioning of EOCs compared to static samples; however, both these groups used heparized blood over a shorter period of time than the work presented here [13], [39]. The TF results due to steady flow preconditioning on EOCs were not consistent with one group observing a decrease in TF gene expression [15], another observing an increase in TF gene expression and protein [40], and a third observing no significant change in TF gene expression [38]; however, as with mature endothelial cells, the application of steady fluid shear stress has generally led to a more anticoagulant phenotype in previous studies of EOCs. While the differences seen with this work could be attributed to minor differences in experimental design such as species, matrix coating, or magnitude of the fluid stress, such profound differences suggest a more fundamental change has occurred.

The largest difference between the previous work and the work presented here is likely the material on which the cells were adhered and the way this material impacted cell morphology, altering the static EOC phenotype. Most of the previous work comparing steady shear to static preconditioning was completed on flat surfaces of matrix-coated tissue culture plastic or glass [14], [15], [36], [37]. The collagen-coated ePTFE grafts used here have a distinct topography with a fiber structure that encourages elongation of the EOCs in the circumferential direction. While EOCs seeded on a truly flat surface will exhibit a primarily cobblestone morphology in the absence of flow, the EOCs in this study were frequently observed to be elongated on the ePTFE without fluid shear stimulation. The significance of this morphological change is the corresponding phenotypic change that accompanies endothelial cell elongation. As reviewed elsewhere [41], endothelial cells that are compelled into an elongated morphology without flow exhibit many similarities to endothelial cells elongated with steady fluid shear stress. Multiple researchers have shown through a direct comparison of endothelial cells elongated with flow or with cell patterning that the extent of elongation and the orientation of the cytoskeleton are very similar between flow and patterned endothelial cells. The transcription factor krüppel-like factor 2, which was previously thought to be shear stress-dependent, has also been shown to increase in patterned endothelial cells, illustrating the importance of elongation on endothelial cell phenotype [42]. Elongation due to either patterning or flow decreased primary endothelial cell expression of both TFPI and von Willebrand factor genes [43], which likely contributes to the similarities seen here in FXa production and platelet accumulation between static and steady flow conditioned EOC samples. Additionally, previous work showed that both methods of elongation lead to a decrease in the expression of inflammation markers such as VCAM and E-selectin [42], offering a potential explanation to the lack of statistical difference in intimal hyperplasia between static and steady shear preconditioned EOC grafts when implanted in an aorto-illiac bypass graft for 1mth. The ePTFE topography contributed to the elongation of the EOCs and the resulting elongated morphology may provide a thromboprotective phenotype similar to that previously observed with steady fluid shear stress. Future work toward altering EOC phenotype through either fluid shear stress or physical cell alignment will be important toward improving antithrombotic function.

In this study, the elongation imparted on the EOCs though the ePTFE ultrastructure resulted in equivalent EOC phenotype for the no flow and steady flow preconditioned cells. However, there was a trend that steady shear stress decreased intimal hyperplasia, when normalized to the contralateral acellular control graft. Additional research should further examine the potential benefit of flow preconditioning of EOC-seeded grafts. Work that seeks to improve biological integration of vascular grafts by harnessing the regulatory function of the native endothelium should be pursued to permit small diameter graft patency. Future work should also identify methods to improve the durability of an intact endothelial layer on graft materials.

Much hope has been placed in the potential to tissue engineer a vascular graft to improve clinical outcomes at small diameters, but constructs that have been created and characterized *in vitro* and subsequently tested *in vivo* with small animal models have yet to translate into a viable graft replacement. For example, work by Stroncek et al. [29]. observed no significant change in intimal hyperplasia when EOCs were transfected with TM compared to unmodified EOCs even though their previous work saw a dramatic increase in APC production with the transfection *in vitro* [44]. While their work did support EOC endothelialization of vascular grafts compared to bare ePTFE grafts, it also motivates a better understanding of the cell phenotype necessary for improving *in vivo* outcomes. Determining the *in vitro* properties that correlate to positive *in vivo* outcomes is essential for the development of successful bioengineered grafts. Combining all of the *in vitro* assessments performed here in parallel to *ex vivo* assessments allowed for a thorough examination of the extent to which these factors directly correlate to platelet accumulation. The second hypothesis of this work was that *in vitro* measures of the EOC functions would correlate with *ex vivo* platelet accumulation. Individually, a variety of *in vitro* metrics of EOC-seeded vascular grafts significantly correlated to platelet accumulation: APC generation, FXa generation, DNA per surface area, and eNOS gene expression. However, none of these individual correlations was very strong. This was not surprising given the multiple regulatory pathways of coagulation, further motivating the combination of factors in a regression model. A multifactor linear regression analysis determined that a model combining FXa generation, eNOS gene expression, and CD39 gene expression correlated well to platelet data. Interestingly, FXa generation was consistently the most predominant of these factors, as was indicated in the statistical analysis by larger beta values, larger t-values, and lower p-values, but expression of genes relating to FXa generation, TF and TFPI, were not significant factors. This suggests the strength of this functional assay compared to gene expression data, which does not reflect post-translational regulation. It was surprising that many of the variables did not significantly correlate to the platelet accumulation, further emphasizing the importance of determining predictive *in vitro* metrics. While many researchers test inflammatory markers, most often using gene expression, the lack of correlation with ICAM and VCAM observed here suggests that inflammation-induced gene expression data may not translate well into clinical performance.

This is the first work of this type, which attempts to determine predictive metrics for *in vivo* vascular graft performance from *in vitro* endothelial markers. The model was created from *ex vivo* platelet data; however, the model of predicted platelet attachment when applied to *in vitro* data from parallel implant samples correlated strongly to intimal hyperplasia. This is a very encouraging result, although not unexpected given the ability of platelets to release growth factors like TGF and PDGF, which encourage smooth muscle cell migration and proliferation—critical to the tissue ingrowth characteristic of intimal hyperplasia [45]–[47]. The strength of this correlation is intriguing and suggests the true potential of these specific *in vitro* tests to predict *in vivo* performance. However, more work is needed to confirm this potential. The study of the ability of EOCs to alter vascular responses *in vivo* is currently being studied with sheep EOCs on tissue engineered grafts [12], [48]. Future work should examine the applicability of our regression model to other tissue engineered graft conditions, including other sources of endothelial cells, graft surfaces, or preconditioning methods. Genetic manipulations to increase single factors within a thromboprotective and thromboprone EC phenotype could identify key regulators of platelet attachment and tissue ingrowth. Having strong predictors for thrombogenicity and ultimately *in vivo* remodeling could drastically decrease the resources necessary to translate a tissue engineered graft into a successful clinical product.

This work presents comprehensive *in vitro*, *ex vivo*, and *in vivo* results on flow preconditioning of EOCs on vascular grafts using steady and oscillatory flow conditions. Additionally, this work addresses an important gap in knowledge by beginning to determine the predictive factors important in graft implant success. Gaining a better understanding of these factors should improve future work on vascular grafts for the long-term, clinical improvement of small diameter vascular graft products.

## Supporting Information

S1 File Raw Data. File contains raw data from all *in vitro*, *ex vivo*, and *in vivo* experiments.
(XLSX)

S1 Fig Representative PECAM staining of EOCs seeded on ePTFE grafts. EOCs were seeded for 24hrs followed by 24hrs of continued static culture (**A**), 10dynes/cm^2^ steady fluid shear stress (**B**), or 0±10dynes/cm^2^ at 1 Hz oscillatory shear stress (**C**). EOCs without flow conditioning showed a cobblestone morphology or random cell alignment. Steady shear stress imparted an elongated cell morphology, with cells aligning in the direction of flow. Oscillatory shear stress induced a rounded morphology. Scale bar equals 200 µm. Arrows indicate the direction of flow stimulation. PECAM was stained red with nuclei in blue.
(TIF)

S2 Fig Actin staining of EOCs seeded on ePTFE grafts for 48hrs of static culture. A majority of EOCs were aligned and elongated in the circumferential direction (top to bottom) along the fiber structure of the ePTFE. Scale bar equals 100 µm.
(TIF)

S1 Table Gene primers for *in vitro* qPCR analysis.
(DOCX)

S2 Table Single variable linear regression analysis. Resulting R^2^ and p-values from the single variable linear regression analysis are shown here. Variables with p-values less than 0.25 were considered during multivariable analysis.
(DOCX)

S3 Table R^2^ and p-values from the linear regression models tested.
(DOCX)

S4 Table Coefficients output from SPSS for selected multifactorial linear regression model.
(DOCX)
