# Supplementary figures and images for: Engineering an Endothelialized Vascular Graft: A Rational Approach to Study Design in a Non-Human Primate Model
Source: PLoS One. 2014 Dec 19;9(12):e115163. doi: 10.1371/journal.pone.0115163 (PMC4272299; doi:10.1371/journal.pone.0115163)

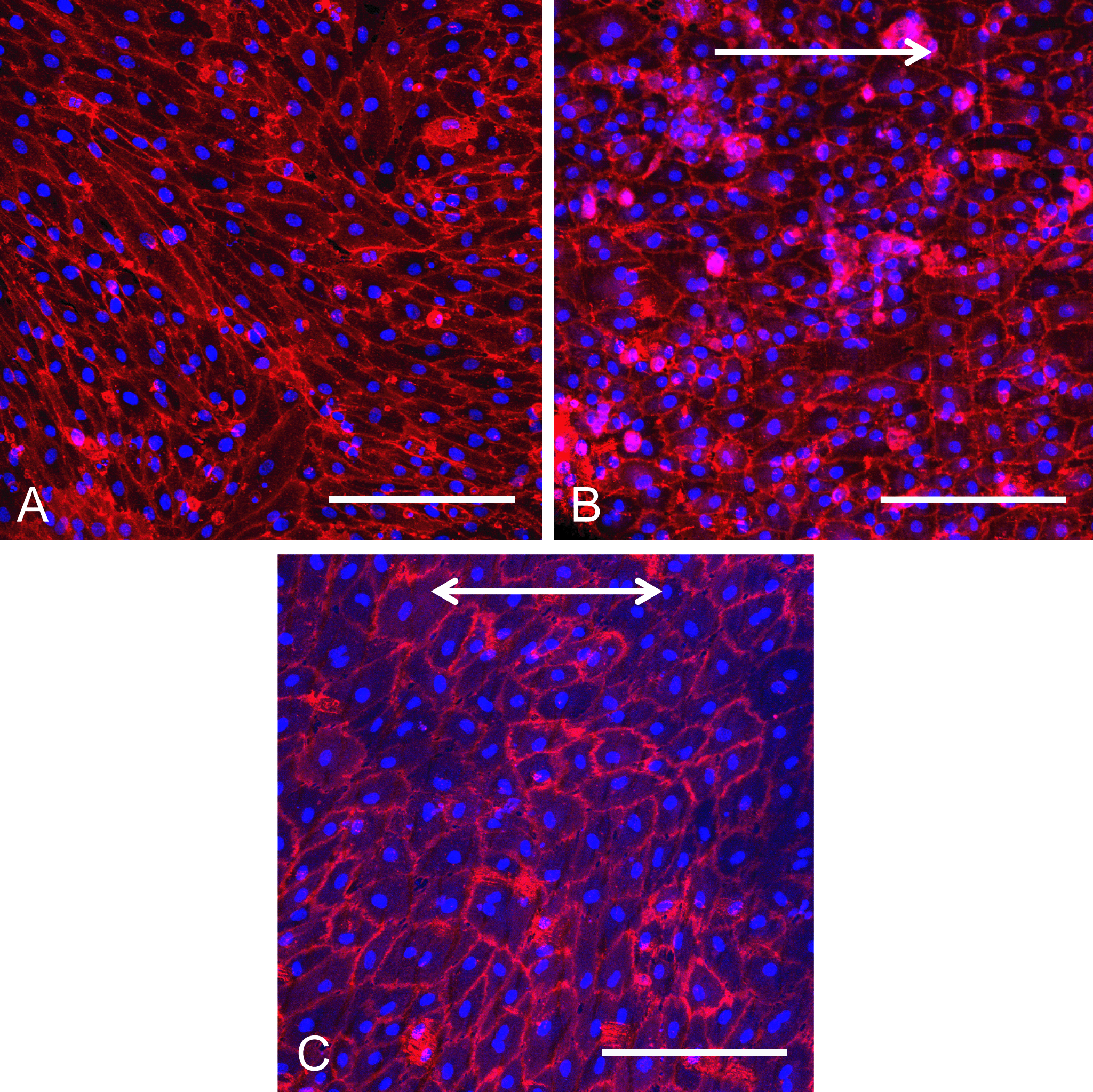

Supplement: S1 Fig — Representative PECAM staining of EOCs seeded on ePTFE grafts. EOCs were seeded for 24hrs followed by 24hrs of continued static culture (A), 10dynes/cm2 steady fluid shear stress (B), or 0±10dynes/cm2 at 1 Hz oscillatory shear stress (C). EOCs without flow conditioning showed a cobblestone morphology or random cell alignment. Steady shear stress imparted an elongated cell morphology, with cells aligning in the direction of flow. Oscillatory shear stress induced a rounded morphology. Scale bar equals 200 µm. Arrows indicate the direction of flow stimulation. PECAM was stained red with nuclei in blue. (TIF) [file pone.0115163.s002.tif]

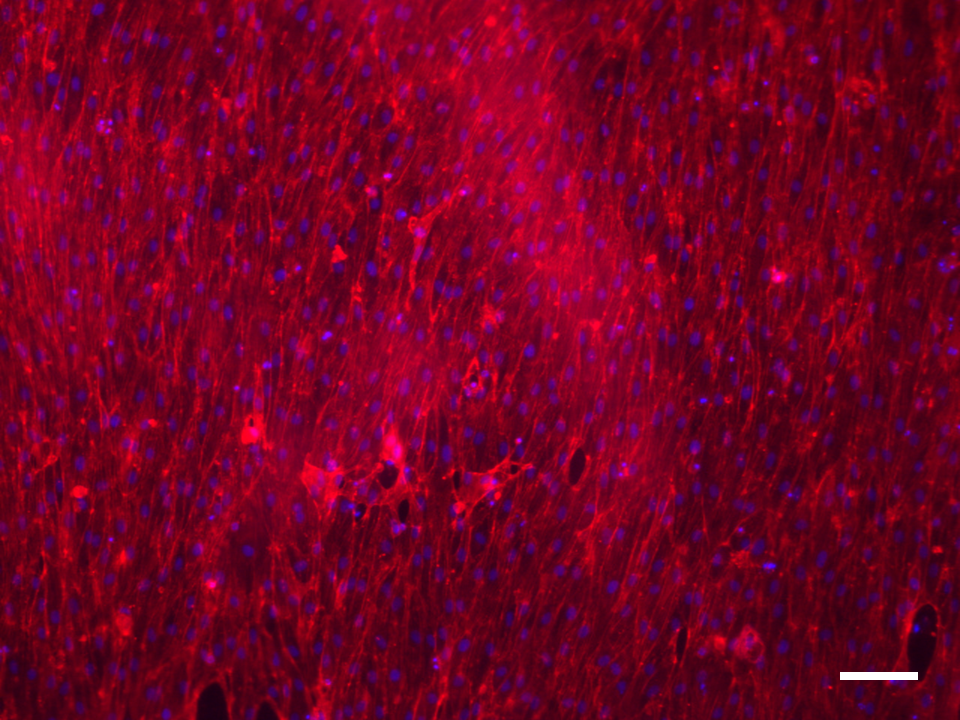

Supplement: S2 Fig — Actin staining of EOCs seeded on ePTFE grafts for 48hrs of static culture. A majority of EOCs were aligned and elongated in the circumferential direction (top to bottom) along the fiber structure of the ePTFE. Scale bar equals 100 µm. (TIF) [file pone.0115163.s003.tif]
