# Supplementary material for: Engineering an Endothelialized Vascular Graft: A Rational Approach to Study Design in a Non-Human Primate Model
Source: PLoS One. 2014 Dec 19;9(12):e115163. doi: 10.1371/journal.pone.0115163 (PMC4272299; doi:10.1371/journal.pone.0115163)
Supplement: S1 Table — Gene primers for in vitro qPCR analysis. (DOCX) [file pone.0115163.s004.docx]

**Table S1. Gene primers for *in vitro* qRT-PCR analysis.**

| **Gene** | **Forward primer** | **Reverse primer** |  |
| --- | --- | --- | --- |
| **GAPDH** | CCTCAACGACCACTTTGTCA | TTACTCCTTGGAGGCCATGT |  |
| **TF** | CACCGACGAGATTGTGAAGGAT | TTCCCTGCCGGGTAGGAG |  |
| **TFPI** | GACTCCGCAATCAACCAAGGT | TGCTGGAGTGAGACACCATGA |  |
| **TM** | GGTGGACGGCGAGTGTGTGG | GGTGTTGGGGTCGCAGTCGG |  |
| **EPCR** | CACCCTGCAGCAGCTCAATGC | ACATCGCCGTCCACCTGTGC |  |
| **CD39** | AGTGATTCCAAGGTCCCAGCACC | TCCTGAGCAACCGCATGCCT |  |
| **eNOS** | TGGTACATGAGCACTGAGATCG | CCACGTTGATTTCCACTGCTG |  |
| **ICAM** | GCAGTCAACAGCTAAAACCTTCCT | GCAGCGTAGGGTAAGGTTCTTG |  |
| **VCAM** | GGGAAGATGGTCGTGATCCTT | TGAGACGGAGTCACCAATCTG |  |
| **PECAM** | CAGCCTTCAACAGAGCCAACC | CACTCCGATGATAACCACTGC |  |
