# Supplementary material for: Engineering an Endothelialized Vascular Graft: A Rational Approach to Study Design in a Non-Human Primate Model
Source: PLoS One. 2014 Dec 19;9(12):e115163. doi: 10.1371/journal.pone.0115163 (PMC4272299; doi:10.1371/journal.pone.0115163)
Supplement: S2 Table — Single variable linear regression analysis. Resulting R2 and p-values from the single variable linear regression analysis are shown here. Variables with p-values less than 0.25 were considered during multivariable analysis. (DOCX) [file pone.0115163.s005.docx]

**Table S2. Single variable linear regression analysis.**

| **Single factors from linear regression** | **R^2^** | **p** |
| --- | --- | --- |
| **DNA** | 0.165 | 0.011** |
| **APC** | 0.196 | 0.005** |
| **FXa** | 0.306 | 0.000** |
| **CD39** | 0.096 | 0.161* |
| **EPCR** | 0.031 | 0.431 |
| **TF** | 0.016 | 0.570 |
| **TFPI** | 0.049 | 0.321 |
| **TM** | 0.088 | 0.181* |
| **eNOS** | 0.203 | 0.035** |
| **ICAM** | 0.003 | 0.815 |
| **VCAM** | 0.001 | 0.915 |
| **PECAM** | 0.126 | 0.258 |

Resulting R^2^ and p-values from the single variable linear regression analysis are shown here.. Variables with p-values less than 0.25 were considered during multivariable analysis.

*p<0.25

** p<0.05
