# Supplementary material for: Engineering an Endothelialized Vascular Graft: A Rational Approach to Study Design in a Non-Human Primate Model
Source: PLoS One. 2014 Dec 19;9(12):e115163. doi: 10.1371/journal.pone.0115163 (PMC4272299; doi:10.1371/journal.pone.0115163)
Supplement: S3 Table — R2 and p-values from the linear regression models tested. (DOCX) [file pone.0115163.s006.docx]

**Table S3.** **R^2^ and p-values from the linear regression models tested.**

| **Factors from linear regression models** | **R^2^** | **p** |
| --- | --- | --- |
| **FXa, eNOS, CD39** | 0.746 | 0.000 |
| **FXa, DNA, CD39, TM** | 0.743 | 0.000 |
| **FXa, APC, CD39, TM** | 0.742 | 0.000 |
| **FXa, CD39, TM** | 0.718 | 0.000 |
| **FXa, APC** | 0.659 | 0.000 |
| **FXa, APC, CD39** | 0.658 | 0.001 |
| **FXa, DNA, CD39** | 0.653 | 0.001 |
| **FXa, CD39** | 0.561 | 0.001 |
| **FXa, eNOS** | 0.552 | 0.001 |
| **FXa, TM** | 0.470 | 0.005 |
| **FXa, DNA** | 0.402 | 0.000 |
| **eNOS, CD39** | 0.351 | 0.016 |
| **APC, CD39, TM** | 0.308 | 0.078 |
| **DNA, CD39, TM** | 0.296 | 0.090 |
| **APC, CD39** | 0.248 | 0.067 |
| **TM, CD39** | 0.247 | 0.067 |
| **DNA, CD39** | 0.224 | 0.089 |
| **APC, TM** | 0.219 | 0.095 |
| **DNA, TM** | 0.187 | 0.140 |
