# Supplementary material for: Engineering an Endothelialized Vascular Graft: A Rational Approach to Study Design in a Non-Human Primate Model
Source: PLoS One. 2014 Dec 19;9(12):e115163. doi: 10.1371/journal.pone.0115163 (PMC4272299; doi:10.1371/journal.pone.0115163)
Supplement: S4 Table — Coefficients output from SPSS for selected multifactorial linear regression model. (DOCX) [file pone.0115163.s007.docx]

**Table S4. Coefficients output from SPSS for selected linear regression model.**

|  | Unstandardized Coefficients | | Standardized Coefficients | t | Sig. |
| --- | --- | --- | --- | --- | --- |
|  | B | Std. Error | Beta |  |  |
| (Constant) | 1.572 | .204 |  | 7.722 | .000 |
| FXa | .367 | .075 | .615 | 4.879 | .000 |
| eNOS | -20.859 | 6.120 | -.431 | -3.409 | .004 |
| CD39 | -187.479 | 53.700 | -.441 | -3.491 | .003 |

Dependent Variable: plateletend
